# Multiscale photoacoustic tomography using reversibly switchable thermochromics

**DOI:** 10.1117/1.JBO.28.8.082804

**Published:** 2023-02-16

**Authors:** Chenshuo Ma, Xiao Kuang, Maomao Chen, Luca Menozzi, Laiming Jiang, Qifa Zhou, Yu Shrike Zhang, Junjie Yao

**Affiliations:** a Duke University, Department of Biomedical Engineering, Durham, North Carolina, United States; b Brigham and Women’s Hospital, Harvard Medical School, Division of Engineering in Medicine, Department of Medicine, Cambridge, Massachusetts, United States; c University of Southern California, Department of Biomedical Engineering and USC Roski Eye Institute, Los Angeles, California, United States

**Keywords:** photoacoustic tomography, reversibly switchable thermochromics, deep tissues

## Abstract

**Significance:**

Based on acoustic detection of optical absorption, photoacoustic tomography (PAT) allows functional and molecular imaging beyond the optical diffusion limit with high spatial resolution. However, multispectral functional and molecular PAT is often limited by decreased spectroscopic accuracy and reduced detection sensitivity in deep tissues, mainly due to wavelength-dependent optical attenuation and inaccurate acoustic inversion.

**Aim:**

Previous work has demonstrated that reversible color-shifting can drastically improve the detection sensitivity of PAT by suppressing nonswitching background signals. We aim to develop a new color switching-based PAT method using reversibly switchable thermochromics (ReST).

**Approach:**

We developed a family of ReST with excellent water dispersion, biostability, and temperature-controlled color changes by surface modification of commercial thermochromic microcapsules with the hydrophilic polysaccharide alginate.

**Results:**

The optical absorbance of the ReST was switched on and off repeatedly by modulating the surrounding temperature, allowing differential photoacoustic detection that effectively suppressed the nonswitching background signal and substantially improved image contrast and detection sensitivity. We demonstrate reversible thermal-switching imaging of ReST *in vitro* and *in vivo* using three PAT modes at different length scales.

**Conclusions:**

ReST-enabled PAT is a promising technology for high-sensitivity deep tissue imaging of molecular activity in temperature-related biomedical applications, such as cancer thermotherapy.

## 1 Introduction

Optical imaging is widely used in life sciences and preclinical research and in clinical applications^1^^,^^2^ but is limited by intense light scattering in tissues, which results in a shallow penetration depth.^3^ Photoacoustic tomography (PAT) overcomes the depth limitation of optical imaging in tissues using acoustic detection of optical absorption. PAT technology has evolved rapidly over the past decades as a novel imaging modality with various biomedical applications^4^^–^^8^ and is particularly well-suited for molecular imaging in deep tissue.^8^^–^^13^ Deep tissue PAT studies have used either endogenous absorbers found in tissue or exogenous contrast agents.^9^^,^^14^ However, the challenge of limited detection sensitivity in deep tissue in the presence of strong background signal has not been adequately resolved. In this study, we demonstrate the use of reversibly switchable thermochromics (ReST) that can improve PAT imaging quality by increasing the contrast-to-noise ratio (CNR).

Chromic materials—dyes or pigments that exhibit a color change when exposed to an external stimulus—have been found to enormously improve the CNR for PAT, especially over the background of abundant endogenous absorbers like hemoglobin in red blood cells.^15^^,^^16^ A variety of chromic materials with different color-changing mechanisms have been developed.^17^^–^^19^ For example, some organic dyes and nanoparticles possess different absorption properties at different concentrations.^20^^,^^21^ Photochromic materials, such as fluorescent proteins that undergo photoinduced electron transfer^22^ and bacterial phytochromes, in which photoisomerization of biliverdin occurs,^23^^,^^24^ exhibit shifts in their absorption spectrum following photoirradiation.^23^^,^^25^ Photochromatic probes are reversibly switchable, easy to use, and can be generically encoded in different tissue types. However, the activation of color change in photochromic probes requires delivering sufficient light energy, which is usually challenging for deep tissues. For example, some well-known small photochromic molecules, such as spiropyrans, can respond only to UV light for color shifting,^26^ which does not allow for deep-tissue applications. Moreover, chromoproteins are usually not commercially available, hindering their broad applications in life sciences. In comparison, thermochromic materials undergo color transitions during heating and cooling cycles within a specific temperature range by different mechanisms.^27^^–^^32^ Inorganic thermochromic compounds change their colors by phase-dependent crystal structure change of the entity, exhibiting thermochromic behavior at temperatures ranging from 70°C to 500°C. By contrast, the molecular structure variations and interconversion of stereoisomeric forms are responsible for the color changes of organic and polymeric thermochromic materials, showing mild and tunable color-changing temperatures. Additionally, thermochromic materials are often encapsulated with film-forming materials to form microcapsules to increase durability and versatility and to provide protection from the external environment. Microencapsulation also protects thermochromic materials from contamination and avoids the melting or sublimation of thermochromic components. Therefore, the organic and polymer-based thermochromic microcapsules (reversibly switchable thermochromic microcapsules [RSTM]) have the advantages of mild-transition temperature, low cost, long durability, and high safety, with broad applications in sensors, coatings, and food packaging.^32^^,^^33^ The color-shifting of RSTM is well-suited for PAT during thermal-related therapy.

In this study, we report the use of water-soluble ReST as a nontoxic chromic contrast agent for multiscale PAT. ReST were fabricated by surface modification of commercial RSTM with the hydrophilic polysaccharide alginate and exhibited excellent dispersion stability, biocompatibility, and reversible color-changing. The absorption spectra of ReST vary with changes in temperature,^33^^–^^35^ leading to changes in photoacoustic intensity. The probes were imaged using three implementations of PAT: (1) second-generation optical-resolution photoacoustic microscopy (SG-OR-PAM),^36^^–^^38^ which images ReST at submillimeter depth and micrometer level resolution; (2) high-speed wide-field photoacoustic microscopy using a cylindrically focused transparent ultrasound transducer (CFT-UT-PAM),^39^ which monitors thermal changes in real time; and (3) photoacoustic computed tomography (PACT) with a linear transducer array,^40^^–^^42^ which provides imaging with large penetration depth and a spatial resolution of hundreds of micrometers *in vitro* and *in vivo*. The results show that ReST exhibit excellent properties for photoacoustic imaging and are promising new contrast agents that improve image quality in photoacoustic molecular imaging.

## 2 Materials and Methods

### 2.1 RSTM Surface Modification

**Fig. 1:**
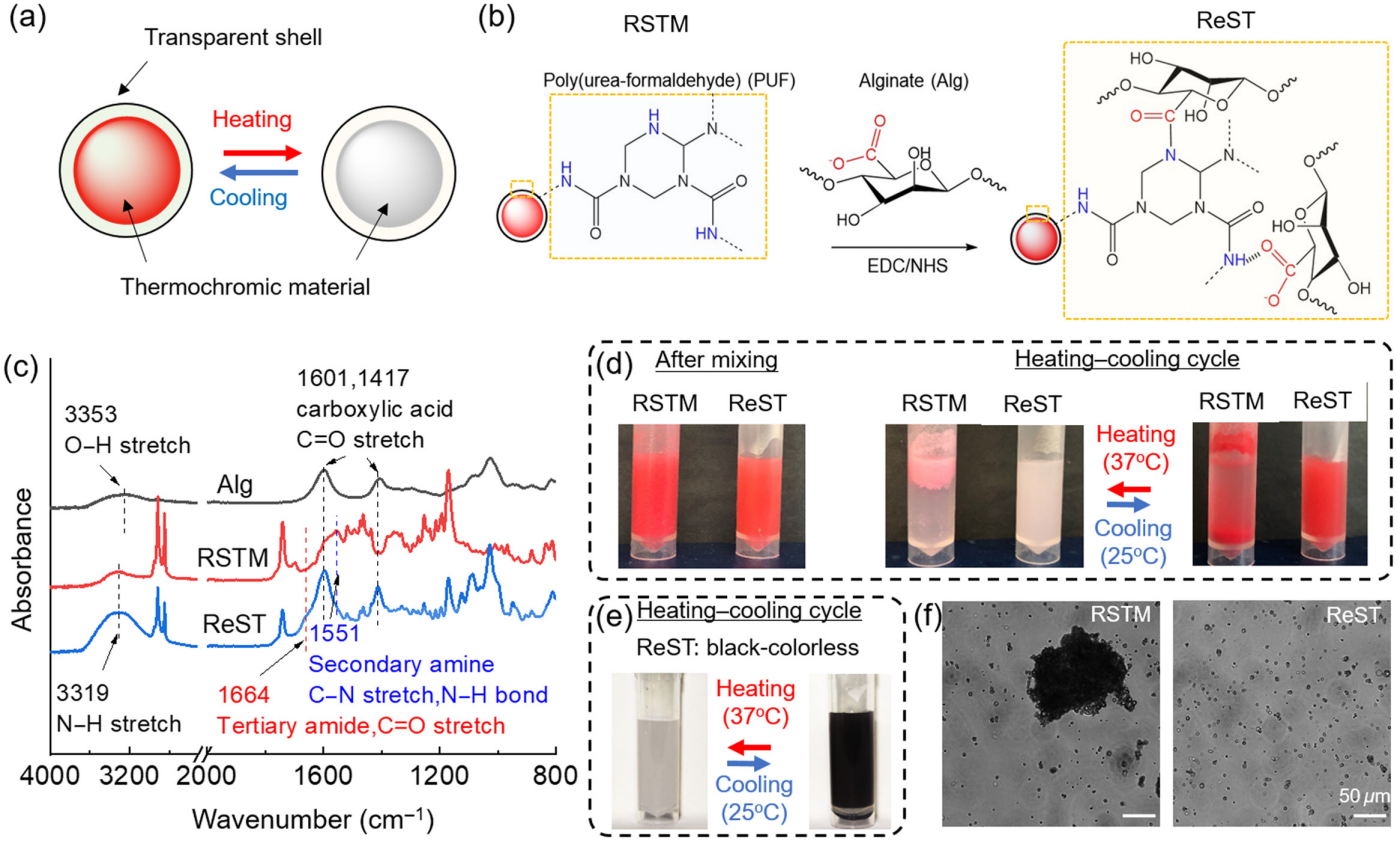
RSTM surface modification: (a) RSTM consist of a thermochromic material surrounded by a transparent hydrophobic polymer shell, and undergo reversible thermal-responsive color changes. (b) Surface modification of an RSTM by alginate (Alg) via EDC/NHS coupling to form a water-dispersible ReST. (c) FTIR spectra of Alg, RSTM and ReST with characteristic bands marked. (d) Reversible color change and stability of aqueous suspensions of red-to-pink RSTM and ReST (2  mg/mL) during a heating-cooling cycle. (e) Reversible color change for aqueous suspension of black-to-colorless ReST (2  mg/mL) during a heating-cooling cycle. (f) Bright-field microscopy images of aqueous dispersions of RSTM and ReST. Scale bars: 50  μm.

Two types of RSTM with different color-shifting modes and transition temperatures ($T_{t}$) were used an RSTM with black-to-white shifting (Atlanta Chemical Engineering) and an RSTM with red-to-pink shifting (LCR Hallcrest: TP31/031CR). RSTM generally contain a thermosetting polymer shell, such as poly(urea-formaldehyde) (PUF)^28^ and chromic material such as a mixture of pigment powder, acid, and solvent. The color change of this system relies on the melting/crystallization of the solvent in the dye mixture without the need for an extra color developer. The solvent is in a solid-state below the crystallization temperature, and the acid binds the dye to give the pigment a red or black color.^43^ Upon heating above the melting temperature, the solvent transforms into a liquid state, and the dye and acid dissociate, resulting in a loss or decrease of color. Thus the RSTM allow safe and reversible color changes [Fig. 1(a)]. However, due to their hydrophobic polymer shell, native RSTM cannot form a homogeneous and stable aqueous suspension and thus are not suitable as contrast agents for PAT.

To address their aqueous insolubility, we modified the surface of RSTM with the biocompatible hydrophilic polymer alginate. The commercially available RSTM consisted of a PUF shell,^28^ and therefore, the polysaccharide alginate could be used to modify RSTM by a reaction between the carboxylic acid groups on alginate chains and secondary amine groups on the RSTM surface [Fig. 1(b)]. Typically, 100 g of 1 wt. % sodium alginate in 2-(N-morpholino) ethanesulfonic acid (MES) buffer (pH 5.5) was activated by 1-ethyl-3-(3-dimethyl aminopropyl) carbodiimide (EDC, $8\times 10^{-4}\;\mathrm{mol}\;L^{-1}$) and N-hydroxysuccinimide (NHS, $8\times 10^{-4}\;\mathrm{mol}\;L^{-1}$) for 30 min at room temperature. Then 1.0 g RSTM dispersed in 40 mL dimethylformamide was added slowly into the solution and the reaction was kept at room temperature for 24 h with gentle stirring. The resulting solution was centrifuged at 5000 rpm and the pellet was washed twice with deionized water. The final solution was dialyzed against deionized water for two days, followed by freeze-drying to obtain a dry powder. All other chemicals were purchased from Sigma-Aldrich and were used as received.

### 2.2 Fourier Transform Infrared Spectroscopy Characterization of ReST

FTIR spectra were recorded on a Nicolet iS50 FTIR spectrometer (Thermo Scientific) from 550 to $4000\;{\mathrm{cm}}^{-1}$ by averaging 32 scans of the signal at a resolution of $2\;{\mathrm{cm}}^{-1}$ in attenuated total reflectance mode. The shell composition and successful surface modification of RSTM were confirmed by FTIR [Fig. 1(c)]. The N─C stretching and N─H bending of secondary amines at $1550\;{\mathrm{cm}}^{-1}$, O─H stretching vibration at $3460\;{\mathrm{cm}}^{-1}$, N─H stretching at 3500 to $3100\;{\mathrm{cm}}^{-1}$, and C═O stretching at $1743\;{\mathrm{cm}}^{-1}$ are assigned to the PUF shell in RSTM. The C═O stretch at 1601 and $1417\;{\mathrm{cm}}^{-1}$ are characteristic bands of carboxylic acid in alginate. After surface modification, the emergence of C═O stretching at $1664\;{\mathrm{cm}}^{-1}$ suggested the formation of a tertiary amide bond, acting as the chemical linkage between RSTM and alginate.

### 2.3 Characterization of ReST Color-Changing Behavior and Stability

Macroscopic color changing and ReST morphology were recorded using a digital camera. The unmodified red-to-pink RSTM in suspension rapidly aggregated after the first heating step [Fig. 1(d)]. In contrast, the modified red-to-pink ReST in suspension remained homogeneous during multiple heating-cooling cycles between 25°C and 37°C and exhibited reversible color changes. Similar behavior was also observed for the modified black-to-colorless ReST [Fig. 1(e)]. Thus the chemically anchored alginate chains on the ReST stabilized the microcapsules without interfering with the color transition of the thermochromic materials inside. Fluorescence microscopy (Eclipse, Nikon) under a bright field validated the excellent dispersion and stability of the ReST suspensions. In contrast to prominent particle aggregation in RSTM suspensions, the microcapsules (3 to 6  μm diameter) in ReST suspensions were evenly distributed in water, as shown by the bright-field microscope and fluorescence microscope images at different magnifications [Fig. 1(f) and Fig. S1 in the Supplementary Material]. Thus ReST exhibited good dispersion stability and reversible color-shifting, making them potentially useful as a chromic contrast agent in PAT.

### 2.4 Optical Absorption Measurements

Absorption spectra were measured using a Shimadzu UV-3600 spectrophotometer with a quartz cuvette. ReST were dispersed in DI water at 2.7  mg/mL. The absorption spectra were measured at 10°C, 20°C, 30°C, 40°C, and 50°C. The excitation wavelength was changed from 350 to 1300 nm with a step size of 1 nm. The temperature-dependent absorption spectra were further analyzed at 532 and 1064 nm. At each wavelength, the solution was heated from 5°C to 50°C with a temperature increase rate of 2°C/min. The solvent (DI water) background was subtracted.

### 2.5 Multiscale PAT

**Fig. 2:**
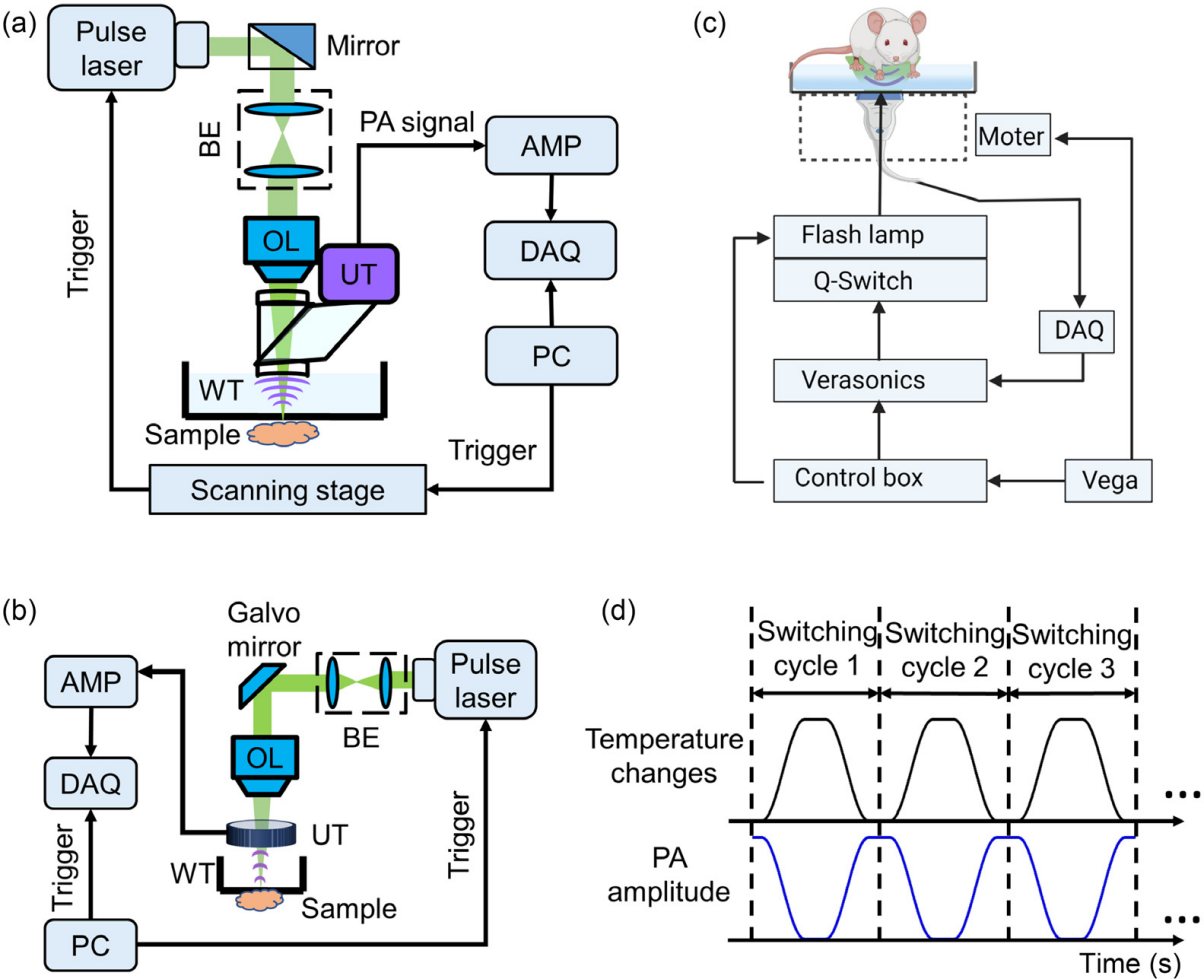
Schematics of the three PAT implementations and the imaging sequence of ReST: (a) SG-OR-PAM, (b) CFT-UT-PAM, and (c) PACT. AMP, amplifier; BE, beam expander; DAQ, data acquisition card; OL, objective lens; UT, ultrasound transducer; and WT, water tank. (d) Time sequences of the thermal switching cycle of ReST.

For SG-OR-PAM [Fig. 2(a)],^37^^,^^38^ excitation was provided by an Nd:YAG fiber laser (IS8II-E, EdgeWave, Würselen, Germany) at 532 nm with a pulse repletion rate (PRR) of 1 kHz. The laser beam was expanded by a beam expander, focused by an objective lens, then delivered to the imaging object. An ultrasound transducer (V214-BB-RM, Olympus-NDT, Massachusetts, USA) detected the resultant photoacoustic signal with a central frequency of 50 MHz. The detected signals were amplified and digitized by the data acquisition card (ATS9350, Alazar Tech, Quebec, Canada) with a sampling frequency of 250 MHz. The optical beam and acoustic wave were confocally aligned by an optical-acoustic combiner, optimizing the imaging sensitivity. A two-dimensional mechanical scanning stage (L-509, PI, Aubum, Massachusetts, USA) provided raster scanning of the sample. The lateral resolution of SG-OR-PAM was 3.7  μm.

For CFT-UT-PAM [Fig. 2(b)],^39^ 532 nm excitation light was provided by an Nd:YAG fiber laser (VPFL-G-20, V-Gen, Tel Aviv, Israel) at a PRR of 250 kHz. The beam expander shaped and expanded the light beam and scanned along the fast-axis direction via a galvanometer scanner (GVS 002, Thorlabs, Newton, New Jersey, USA). The scanning beam was focused by an objective lens with a focal length of 50 mm, transmitted through the CFT-UT, and delivered to the imaging target. The acoustic focal line of the CFT-UT was confocally aligned with the optical focus along the scanning direction (fast axis) of the galvanometer scanner. At the same time, the slow axis was mechanically scanned by a motorized stage (L-509, PI, Aubum, Massachusetts, USA). The resultant photoacoustic signal was detected by the CFT, amplified, and digitized by the data acquisition card (ATS9350, Alazar Tech, Quebec, Canada) with a sampling frequency of 250 MHz.

For PACT [Fig. 2(c)], experiments were performed using a hybrid imaging system for PACT and high-frequency ultrasound imaging.^40^^–^^42^ The excitation source was a 532-nm pulse laser (Q-smart 850, Quantel Laser, MT, USA), and the PRR was 10 Hz. The excitation light was coupled into a two-branch linear fiber bundle that flanked the linear transducer array and guided to the target surface. The generated photoacoustic signals were received by a 128-element linear array ultrasound transducer (Philips L7-4, Massachusetts, USA; central frequency, 5 MHz,). The system’s spatial resolution is ∼300  μm along the axial and lateral directions and ∼1  mm along the elevational direction. A high-frequency wobbler ultrasound transducer was used to acquire the B-mode ultrasound images with a central frequency of 35 MHz.

Figure 2(d) shows sequences of ReST thermal switching cycles. In each cycle, the temperature was changed between 20°C and 40°C. For all PAT experiment, 532-nm light illumination was used. PA images were acquired at different temperature points using SG-OR-PAM and PACT imaging, and continuous real-time imaging was performed using CFT-UT-PAM.^39^

### 2.6 *In Vitro* Multiscale PAT of ReST

For *in vitro* studies using SG-OR-PAM and CFT-UT-PAM, ReST were dispersed in DI water, and a “D” shape pattern was deposited using the red ReST suspension on a glass slide. After the water evaporated, a thin layer of ReST remained on the slide for imaging. For *in vitro* studies using PACT, the red or black ReST were mixed with Matrigel and implanted in an agar phantom. One agar phantom with the red ReST contained 7 wt. % agar and 5 vol. % intralipid. Two additional agar phantoms with black and red ReST contained 7 wt. % agar, 5 vol. % intralipid, and 10 vol. % bovine blood to provide the background signals.

### 2.7 *In Vivo* PACT of ReST

Healthy Swiss Webster mice (12 weeks, Hsd: ND4, Envigo.) were used for *in vivo* studies. The protocol for animal experiments was approved by the Institutional Animal Care and Use Committee of Duke University. Mice were fully anesthetized throughout the imaging experiments using 1.5% isoflurane gas mixed with air. The hair on the left leg or lower flank of the mouse was removed, and 100  μL of Matrigel-mixed red or black ReST were injected subcutaneously in the mouse leg or flank, mimicking a solid tumor targeted by ReST. The ReST-injected region was subjected to three heating-cooling cycles from 20°C to 40°C performed using cold and warm water and imaged by the hybrid PACT and ultrasound imaging system. Temperature was monitored with a needle thermometer (Fisherbrand, Traceable, 1500714).

## 3 Results

### 3.1 Optical Absorption Properties of ReST

**Fig. 3:**
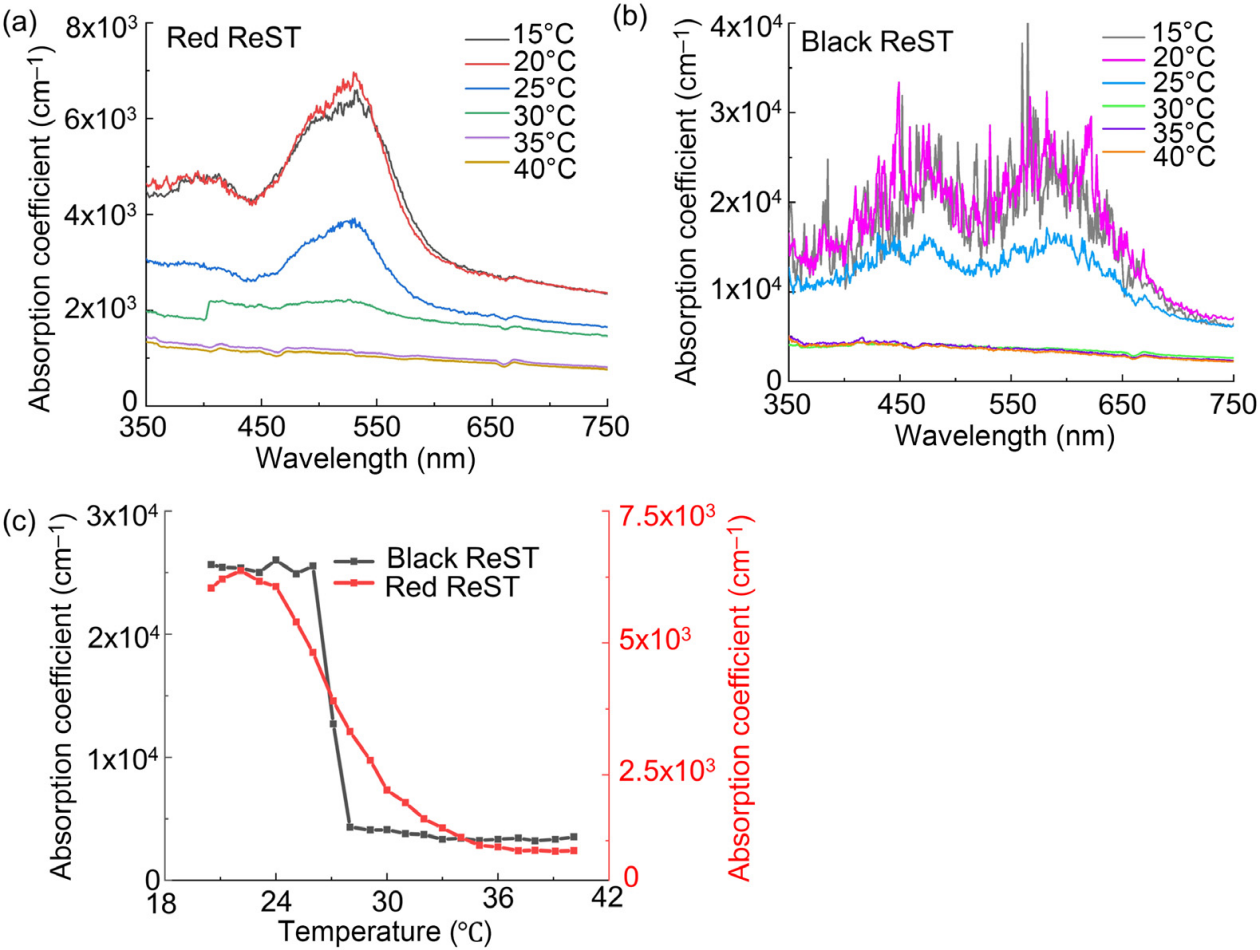
Absorption characterization of ReST. Absorption spectra of (a) red-to-pink ReST (“red ReST”) and (b) black-to-colorless ReST (“black ReST”) at 15°C, 20°C, 25°C, 30°C, 35°C, and 40°C. (c) Temperature-dependent absorption of red (right y axis) and black (left y axis) ReST at 532 nm from 20°C to 40°C.

Figure 3(a) shows absorption spectra of the red-to-pink ReST (“red ReST”) at different temperatures from 15°C to 40°C. The absorption peak at 525 nm is unchanged between 15°C and 20°C and is reduced by 57% upon heating to 25°C. No prominent absorbing peak was observed at 30°C or higher. Figure 3(b) shows absorption spectra of the black-to-colorless ReST (“black ReST”). The absorption peaks at 460 and 590 nm were unchanged between 15°C and 20°C and were reduced by 53% upon heating to 25°C. No prominent absorbing peak was observed at 30°C or higher. Figure 3(c) shows the red and black ReST absorption coefficient versus temperature. For the black ReST, the absorption coefficient was high below 26°C and decreased rapidly (by 85%) between 26°C and 28°C. The red ReST have a 70% lower absorption coefficient than the black ReST below 26°C, and a slower absorption decreases with increasing temperature. Based on the absorption characterization results, both red and black ReST are suitable for thermal reversible photoacoustic imaging with 532 nm excitation. The black ReST are better for deep-penetration PACT due to their higher absorption coefficient.

### 3.2 High-Resolution PAM Imaging of ReST

**Fig. 4:**
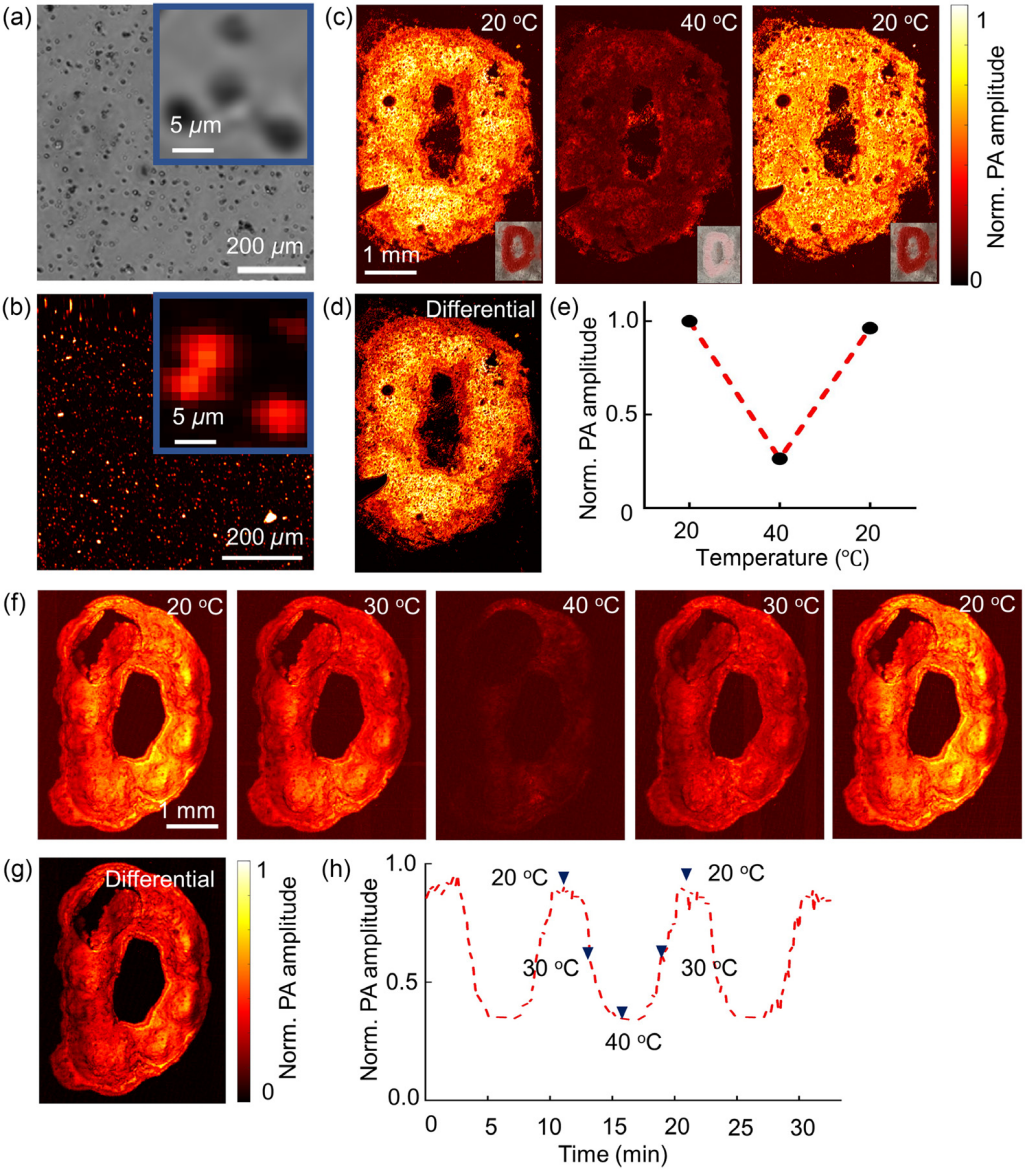
High-resolution PAM of ReST: (a) bright-field microscopy image of dispersed red ReST at 2  mg/mL. Scale bar: 200  μm. Inset: close-up bright-field microscopy image of single red ReST capsules. Scale bar: 5  μm. (b) SG-OR-PAM MAP image of dispersed red ReST. Scale bar: 200  μm. Inset: close-up SG-OR-PAM MAP image of single red ReST capsules. Scale bar: 5  μm. (c) SG-OR-PAM MAP images of the “D” shaped red ReST pattern, imaged at 20°C, 40°C, and 20°C. Inset: photographs of ReST pattern at the corresponding temperature. (d) Differential photoacoustic image between images at 20°C and 40°C. (e) Normalized photoacoustic amplitude in (c). (f) CFT-UT-PAM MAP imaging of red ReST at 20°C, 30°C, 40°C, 30°C, and 20°C. (g) Differential photoacoustic image between images at 20°C and 40°C. (h) Averaged photoacoustic signal amplitude change during temperature changes for the red ReST (Video 1, MOV, 6.477 MB [URL: https://doi.org/10.1117/1.JBO.28.8.082804.s1]).

We report two different PAM systems to demonstrate the feasibility of high-resolution photoacoustic imaging of ReST. SG-OR-PAM was used to image the red ReST at micrometer level spatial resolution.^36^ The lateral resolution of SG-OR-PAM was 3.7  μm, which was smaller than the red ReST particle size (∼5  μm). Figure 4(a) shows an image of dispersed red ReST by bright-field microscopy; Fig. 4(b) shows the corresponding maximum amplitude projection (MAP) image by SG-OR-PAM at the single-particle level.

We first imaged a ‘D’-shaped red ReST phantom. Figure 4(c) shows the photoacoustic images at different temperatures. The baseline image was acquired at 20°C with strong photoacoustic signal detected. When the sample was heated to 40°C, its color changed to light pink, and the photoacoustic signal decreased dramatically. After reducing the phantom temperature back to 20°C, the sample color changed back to red, and the photoacoustic signal amplitude recovered to over 90% of the baseline value. These results demonstrated the excellent reversibility of the ReST for photoacoustic molecular imaging. In this study, the speed of sound in the tissues increased ∼2% from 20°C to 40°C since the speed of sound depends on the temperature. The imaging resolution may be degraded when a constant speed of sound was applied in the image formation but should be a small affect compared with the change in the optical absorption of the ReST (∼1000%). Further, we generated a differential photoacoustic image between 20°C and 40°C [Fig. 4(d)], which can remove any nonswitching signals and improve the image contrast.

In photoacoustic molecular imaging, it might be necessary to detect the molecular probes in real time. To this end, the thermal switching of red ReST was monitored using a high-speed CFT-UT-PAM at a frame rate of 0.2 Hz (5 s per image) to demonstrate the possibility of real-time photoacoustic molecular imaging as well as the repeatability of the red ReST color changes (shows in Video 1). The D-shaped sample was continuously imaged for 32.5 min, with baseline images acquired at 20°C for the initial 2.5 min, followed by three heating-cooling cycles. In each cycle, the sample was heated from 20°C to 40°C within 2.5 min, kept at 40°C for 2.5 min, and cooled from 40°C to 20°C within 2.5 min. Five representative images at different temperatures within one heating-cooling cycle were shown to demonstrate the photoacoustic dynamics of the red ReST [Fig. 4(f)]. The quantitative results are shown in Fig. 4(h). Strong photoacoustic signals were detected [marked by the black triangle in Fig. 4(h)] when the sample was at 20°C due to the strong absorption of the ReST. Then the photoacoustic signal amplitude dropped significantly at 13.5 min when the sample was heated to ∼30°C. At 16 min, when the temperature reached 40°C, the ‘D’ shaped phantom was barely visible [Fig. 4(f)]. The photoacoustic signal amplitude at 40°C was ∼30% of that at 20°C. Figure 4(g) shows a differential image between 20°C and 40°C. At 21 min, the photoacoustic amplitude recovered to the baseline level following cooling back to 20°C. The photoacoustic dynamics illustrate that the temperature-induced color changes of the ReST could be detected in real time.

### 3.3 ReST Reversibility in PACT

**Fig. 5:**
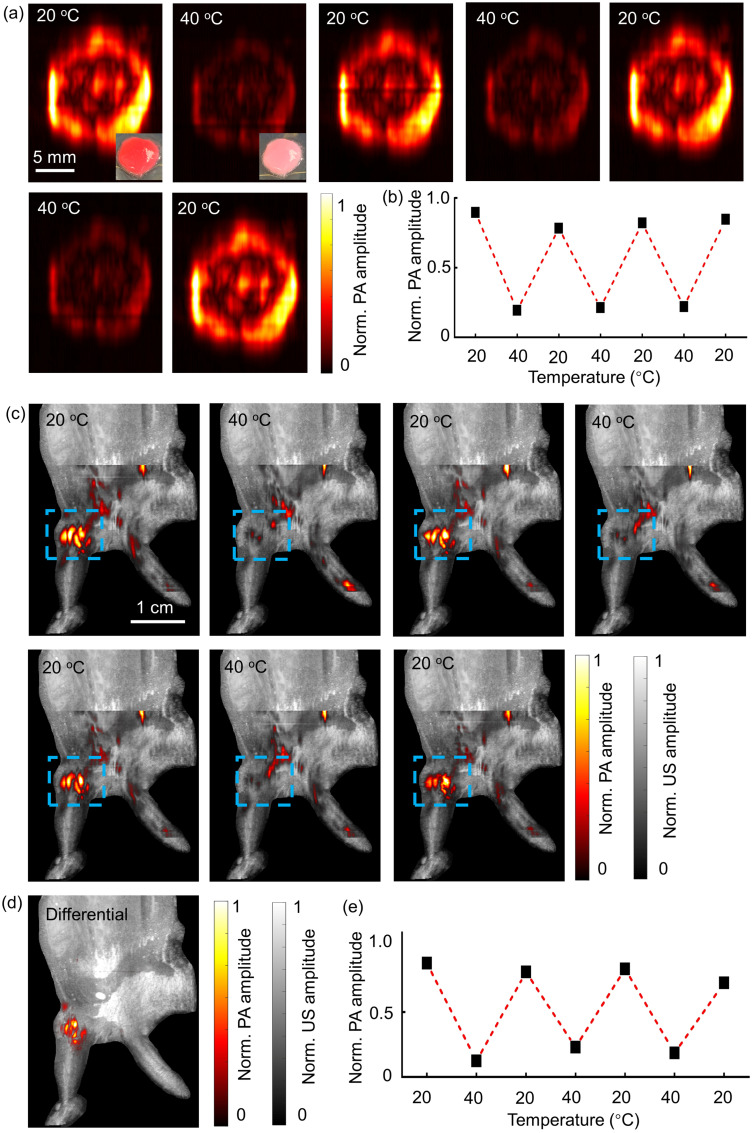
PACT of red ReST *in vitro* and *in vivo*: (a) *in vitro* PACT images of red ReST mixed with Matrigel phantom cycling between 20°C and 40°C. (b) Normalized photoacoustic signal amplitude at different temperatures, corresponding to the photoacoustic images in (a). (c) *In vivo* PACT images of the red ReST-Matrigel implant in the mouse leg at 20°C and 40°C for three cycles, overlayed with ultrasound image of mouse anatomy. (d) Differential photoacoustic image between images at 20°C and 40°C. (e) Normalized photoacoustic amplitude versus temperature. Each data point corresponds to the average photoacoustic amplitude in (c).

We selected the red ReST to demonstrate its color change reversibility and utility in PACT *in vitro* and *in vivo*. A phantom was subjected to three heating-cooling cycles from 20°C to 40°C and then back to 20°C. PACT images are shown in Fig. 5(a). Strong photoacoustic signals were detected due to the high absorption at 20°C. After heating the sample to 40°C, the photoacoustic signal amplitude decreased dramatically. When the sample temperature was cooled back to 20°C, the photoacoustic signals recovered. The temperature-dependent photoacoustic signal amplitude of the ReST was quantitatively analyzed [Fig. 5(b)]. The photoacoustic amplitude at 20°C was about four times higher than that at 40°C. After three heating–cooling cycles, the photoacoustic signal amplitude returned to a similar level as the baseline, demonstrating the reversibility and photostability of the ReST.

Figure 5(c) shows *in vivo* experimental result of the overlayed ultrasound and photoacoustic images at different temperatures. The ultrasound images provide mouse anatomy, whereas the photoacoustic images monitor the ReST color changes. When the mouse leg was immersed in 20°C water, the ReST (highlighted by the dashed blue box) show a strong photoacoustic signal. The ReST signal was much stronger than the photoacoustic signal of the surrounding blood vessels. After increasing the temperature of the water to 40°C, the photoacoustic signal of ReST injection area decreased to ∼25% of the baseline. When the water temperature was reduced back to 20°C, the ReST photoacoustic signal returned to baseline. We also generated a differential PA image between 20°C and 40°C [Fig. 5(d)], which removed any nonswitching signals and improved the image contrast. Photoacoustic images were acquired for another three heating–cooling cycles, and the temperature-dependent photoacoustic signal amplitude was quantified [Fig. 5(e)]. The results demonstrate three cycles of reversible switching between 20°C and 40°C, illustrating the reversibility of ReST in PACT.

### 3.4 Deep Tissue PACT of ReST with Improved Image Contrast

**Fig. 6:**
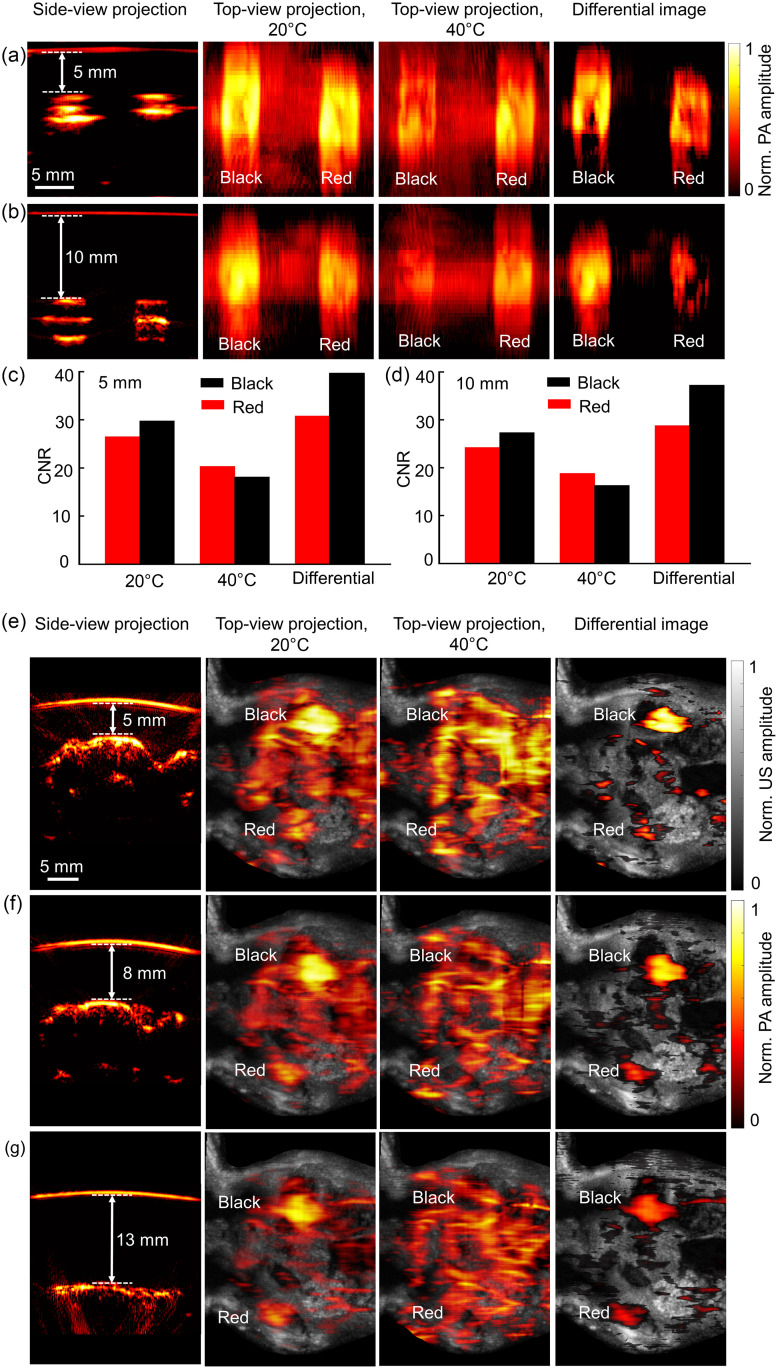
Deep-tissue PACT of the red and black ReST. (a), (b) *In vitro* PACT projection images of red- or black-ReST-Matrigel phantom at 5 and 10 mm depth at 20°C and 40°C, as well as the differential photoacoustic image between 20°C and 40°C. All top-view photoacoustic projection images are shown in log scale. (c), (d) Comparison of CNR quantified from images at different temperatures as well as the differential image at 5 and 10 mm depth. (e)–(g) *In vivo* PACT projection images of the ReST-Matrigel implant in mouse flank at 5, 8, and 13 mm depth, as well as the corresponding differential photoacoustic images.

We further tested the application of ReST in deep tissue PACT to suppress the background signals and improve the image contrast by taking the differential image at high and low temperatures. First, phantoms with both red and black ReST mixed Matrigel at 5-mm depth [Fig. 6(a)] and 10-mm depth [Fig. 6(b)] were imaged at 20°C and 40°C. Strong photoacoustic amplitude was detected at 20°C for both black and red ReST with 5-mm depth phantom. After heating the phantom to 40°C, the photoacoustic amplitude of both the black and red ReST decreased, more so for the black ReST. In the differential image, the black ReST signal was stronger than the red ReST, and the nonswitching background signal was weaker than that in the original photoacoustic images acquired at 20°C and 40°C. The 10-mm-deep phantom demonstrated a similar temperature-related trend as the 5-mm-deep phantom, but the photoacoustic signal amplitudes were lower due to the light attenuation [Fig. 6(b)].

The differential images had higher CNR than the original images, and the black ReST had higher CNR than the red ReST, shown in Figs. 6(c) and 6(d). For red ReST in the 5-mm-deep phantom, the improvement in CNR was 16.2% versus the image at 20°C, and 51.6% versus the image at 40°C. For the black ReST, the improvement in CNR was 33.2% versus the image at 20°C and 119.1% versus the image at 40°C. Similar improvements in CNR by the differential images were obtained on the 10-mm-deep phantom.

For the *in vivo* experiments with the ReST-Matrigel implant in the mouse flank, we overlayed blood-mixed agar with different thicknesses (5, 8, and 13 mm) on top of the mouse skin to mimic different imaging depths, shown in Figs. 6(e)–6(g). The photoacoustic images were presented in log scale to better depict the weak signals, whereas the ultrasound images were shown in linear scale. At 5-mm depth, the background signal amplitude from blood vessels was strong at both 20°C and 40°C [Fig. 6(e)]. With the strong background signals, we were not able to visualize the red ReST implant at 20°C or distinguish black and red ReST implants at 40°C. In contrast, the differential photoacoustic image clearly showed the black and red ReST implant location, with much reduced background blood signals. Additional imaging was performed at 8- and 13-mm depth [Figs. 6(f) and 6(g)]. The differential images at all depths clearly improved the contrast of the ReST implants, even though the photoacoustic signal amplitude decreased with imaging depth due to light attenuation.

## 4 Discussion

In this study, we report a multiscale PAT method using water-soluble ReST to overcome the challenges of reduced detection sensitivity in the presence of strong background signals in deep tissues. We demonstrate that ReST—created by modifying commercial hydrophobic thermochromic microcapsules with the hydrophilic polysaccharide alginate—exhibit excellent photostability and fast reversible color change in response to temperature changes.

The performance of the ReST was comprehensively studied using various PAT techniques. First, the red ReST was imaged with high spatial resolution using SG-OR-PAM, which may be used for detection of the thermal heterogeneity within different areas of a target. The SG-OR-PAM experiments demonstrated that the ReST is a highly absorbing contrast agent for PAT with repeated thermal switching. Second, ReST exhibited a fast response to temperature changes, as imaged by CFT-UT-PAM. This fast temperature responsiveness makes it possible to monitor thermal changes in real time. Third, we also found that ReST could be detected in deep tissue regions *in vivo* using PACT at depths of more than 10 mm. More importantly, the differential images of ReST improved the CNR, by eliminating strong nonswitching background signals from blood vessels.

We have taken several measures to improve the biosafety of our RSTMs. First, the commercially available RSTMs are mostly made of microcapsules consisting of thermochromic cores in an inert and stable PUF shell. The thermochromic materials may undergo color transitions in response to temperature changes over a wide temperature range. Here we specifically chose the RSTM candidates that respond within a temperature range around the body temperature. Second, we have modified the microcapsules using a biocompatible and hydrophilic polymer of alginate. The surface modification enables excellent water dispersion capability of RSTMs and enhances their biosafety. Third, during our *in vivo* experiments, the mouse leg was immersed in water, and the tissue temperature was changed by adjusting the water temperature. The water temperature increase to 40°C was temporary and should not induce any damage to the tissues.

We expect that the technique described in this work provides the foundation for using ReST as a photoacoustic molecular probe, but additional work is needed to further develop ReST for more preclinical applications. First, we introduced systematic temperature changes in our experiments by heating or cooling the immersion water, but spatially confined temperature change is preferred. There are different ways of modulating local temperatures, such as photothermal treatment,^44^^,^^45^ high-intensity focused ultrasound (HIFU) heating treatment, and microwave.^13^^,^^46^^–^^48^ Second, we used ReST as a nontargeting contrast agent in our experiments. More efforts should be invested to develop the targeting capabilities of the ReST. For example, the ReST particle can be conjugated with antibodies to target tumor cell surface receptors and accumulate in tumor tissue.^49^^,^^50^ Third, the red and black ReST used in our experiments are both absorbing in the visible light region. To image ReST at greater depths, near-infrared absorbing chromic molecules or bioactive polymers needs be developed to fabricate ReST.^40^^,^^51^^,^^52^ Fourth, it is challenging to perform the temperature modulation in the brain tissues using our current experimental setup. For future applications on the brain, we may introduce the local temperature change during laser interstitial thermal therapy and HIFU-based thermal therapy. Fifth, we demonstrate the reversible color change in response to temperature changes from 20°C to 40°C, but we did not focus on fine-tuning the transition temperatures of the RSTMs. We envision that thermochromic materials can be further engineered to satisfy fine-tuned temperature transition for different life science applications. For example, we can choose Leuco dyes to achieve color-to-colorless transition in thermochromic microcapsules.^30^ The transition temperature of RSTMs can be fine-tuned by altering the crystallization/melting temperature of the dispersion solvent, as the crystallization and melting temperatures of the solvent determine the temperature in which the color transformation happens.

In summary, deep-tissue molecular PAT with high sensitivity is an area of intense research. Here we have used various PAT systems to demonstrate the feasibility of using water-soluble reversibly switchable thermochromic to improve photoacoustic image contrast. We expect that ReST-enabled PAT will provide a valuable platform for reversibly switchable molecular imaging and can be expanded to a broad range of biomedical applications.
