# Supplementary material for: Multiscale photoacoustic tomography using reversibly switchable thermochromics
Source: J Biomed Opt. 2023 Feb 16;28(8):082804. doi: 10.1117/1.JBO.28.8.082804 (PMC9932525; doi:10.1117/1.JBO.28.8.082804)

Supporting information

Supplementary Video 1. High-speed PAM of the red ReST signal change during three heating-cooling cycles.

Supplementary Figure S1. Bright-field microscope and fluorescence microscope images of aqueous dispersions of RSTM (top) and ReST (bottom) at different magnifications. Scale bars are labeled.

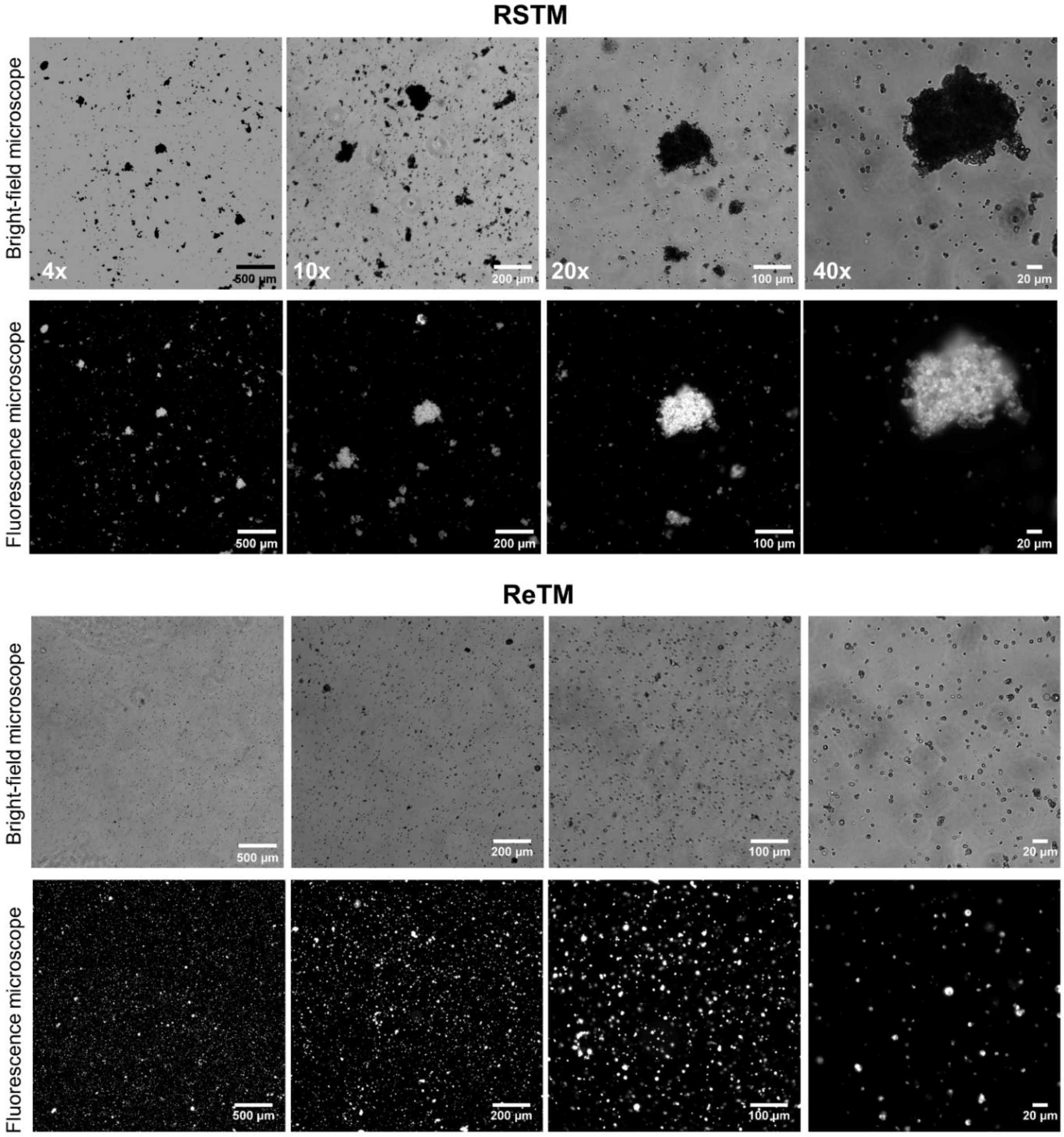

Supplement: Supplementary file 1 [file JBO_028_082804_SD001.pdf]
